# Safety and results of image-guided vertebroplasty with elastomeric polymer material (elastoplasty)

**DOI:** 10.1186/s41747-018-0062-5

**Published:** 2018-10-24

**Authors:** Giovanni Mauri, Luca Nicosia, Luca Maria Sconfienza, Gianluca Maria Varano, Paolo Della Vigna, Guido Bonomo, Franco Orsi, Giovanni Carlo Anselmetti

**Affiliations:** 1 Department of Interventional Radiology, European Institute of Oncology, 20141 Milan, Italy; 2 Postgraduation School in Radiodiagnostics, Università degli Studi di Milano, Facoltà di Medicina e Chirurgia, via Festa del Perdono, 7, 20122 Milan, Italy; 3 Unit of Diagnostic and Interventional Radiology, IRCCS Istituto Ortopedico Galeazzi, Via R. Galeazzi 4, 20166 Milan, Italy; 4 Department of Biomedical Sciences for Health, University of Milano, Via Pascal 36, 20135 Milan, Italy; 5 GVM Care&Research, Maria Pia Hospital, Strada Mongreno 180, 10132 Turin, Italy

**Keywords:** Pain, Pain management, Radiology (interventional), Silicone (elastomers), Vertebroplasty

## Abstract

**Background:**

Image-guided elastoplasty is an innovative method for percutaneous vertebral augmentation with a silicone elastomeric material. Our aim was to evaluate its technical success, safety and efficacy as well as the rate of secondary fractures.

**Methods:**

Nineteen patients (13 women and 6 men, age 72 ± 10 years, mean ± standard deviation) underwent elastoplasty between 2010 and 2016. A total of 33 vertebrae were treated. A total of 2–6 mL of silicone-based elastomeric polymer material (VK100) was used. Visual analogue scale (VAS) and Oswestry disability index (ODI) pain scores were used.

**Results:**

In all cases, it was possible to complete the procedure (technical success 100%). No major complications occurred. In 6/19 (31.5%) patients, asymptomatic leakage of the material was observed during the procedure. Full pain recovery was obtained in 18/19 (94%) patients. One patient with a painful angioma did not experience any change in symptoms. VAS and ODI were significantly reduced after the procedure, from 7.9 ± 1.1 to 0.7 ± 1.4 and from 79.6 ± 12% to 9.9 ± 14% respectively (*p* < 0.001 for both comparisons). After vertebroplasty, 14 of 15 patients (93%) removed the brace and 16/19 (84%) completely stopped using any drugs for pain relief (*p* < 0.001 for both pre-procedure versus post-procedure comparisons). At a mean follow-up time of 26.5 ± 28.1 months (median 8.7 months, range 6–69 months), no secondary fracture occurred.

**Conclusion:**

Taking into consideration the relatively small sample size, image-guided elastoplasty seems to be a safe procedure providing effective pain control over time.

## Key points

- Elastoplasty is an innovative method for vertebral augmentation with a silicone elastomeric material.
- In a series of 19 patients, elastoplasty was a safe and effective procedure.
- No symptomatic leakage of elastomeric material or cases of pulmonary embolism were observed.

## Background

Percutaneous vertebral augmentation is increasingly used to achieve vertebral stabilisation and pain relief from symptomatic vertebral lesions. With this technique, the stiffness of the vertebral body is rapidly restored, reporting significant pain relief and improvement in quality of life [1–4]. The most widely used method for vertebral augmentation is vertebroplasty, which involves injection of surgical bone cement. To this aim, polymethylmethacrylate (PMMA) [5] presents a good characteristic in terms of viscosity, polymerisation time and mechanical resistance. However, PMMA does have some shortcomings and is used primarily because biomaterials available for vertebroplasty were, until recently, limited to PMMA. Among the most feared complications of vertebroplasty is the possible leakage of material, which may damage surrounding tissue and cause nerve compression with pain, spinal cord compression, and even pulmonary embolism [1, 4]. Moreover, as PMMA could be an irritant for soft tissue and is harder and stiffer than natural bone, researchers have postulated that the use of this material might increase the risk of adjacent vertebral fractures [6].

Therefore, the use of a silicone elastomeric material (VK100) has been proposed to achieve vertebral stabilisation with a pliable material that has a modulus closer to that of bone, thus theoretically reducing the risk of adjacent fractures [7]. However, VK100 being less viscous than PMMA, concerns exist due to a theoretical increased risk of leakage. There are only a few reports regarding the application of VK100 to perform vertebral stabilisation, so-called ‘elastoplasty’, with different results. Urlings and van der Linden reported 60% of pulmonary embolism in a series of 12 patients, concluding that silicone material should not be used for the treatment of vertebral fractures [8]. In a different study comparing VK100 and PMMA for vertebral stabilisation after balloon kyphoplasty in a group of 30 patients, no significant difference was found in material embolisation rates [7].

Thus, the primary aim of our work was to investigate the technical feasibility and safety of VK100 elastoplasty. The secondary aim was to assess pain management and the rate of secondary fractures following VK100 elastoplasty.

## Methods

### Patient population

Institutional review board approval was obtained and patient informed consent was waived for this retrospective study. From 2003 to 2016, 4580 patients with back pain and different kinds of vertebral lesions were presented to the same interventional radiologist with more than 13 years of experience; they were treated by percutaneous vertebral augmentation at different institutions. Out of those patients, 19 (0.4%, 13 women and 6 men, age 72 ± 10 years, mean ± standard deviation) underwent elastoplasty in a single institution. The indication for the treatment was the same as for standard vertebral augmentation, which has been previously reported [9–11].

Patients with no more than two vertebral fractures were selected for elastoplasty and prepared for treatment at one of the institutions where the material was available. Fourteen patients had osteoporotic fractures, two patients had traumatic fractures, one patient had a painful myeloma localisation and one patient had a painful vertebral non-aggressive angioma. Fifteen patients used a brace and all were using drugs for pain relief. A total of 33 vertebrae were treated (range T6–L5). Data of all treated patients were prospectively collected in a dedicated database and retrospectively extracted and analysed.

### Technique

Elastoplasty was always performed using a sterile technique in conjunction with prophylactic intravenous antibiotics (2 g of Ceftriaxone) in a dedicated angiosuite equipped with flat-panel digital fluoroscopy (Allura Xper CT; Philips, the Netherlands). The procedure was always performed with the anaesthesiologist in the room. Based on the patient’s condition, it was decided whether to perform the procedure under sedation (Midazolam, 2–5 mg) or general anaesthesia. Vital signs (heart rate, pulse oximetry and blood pressure) were monitored throughout the whole procedure. Under fluoroscopic guidance, a 22-gauge Quincke needle was placed over the pedicle periosteum and local anaesthesia with ≤ 2 mL of 2% lidocaine hydrochloride was administered. Bevelled access cannulas (13 G, Optimed, Ettlingen, Germany) were inserted using the oblique projection and subsequently advanced in the anteroposterior projection. Once the desired position of the needles was achieved and confirmed, 2–6 mL of silicone-based elastomeric polymer material (VK100, BŌNWRx Ltd., Lansing, MI, USA) were gently manually injected under continuous fluoroscopic monitoring.

VK100 is composed of two paste components mixed during application. Component A is composed of dimethyl methylvinyl siloxanes (86%), barium sulfate powder (14%) and a platinum catalyst (15 ppm as metal). Component B is composed of dimethyl methylvinyl siloxanes (78%), barium sulphate powder (15%) and a methylhydrogensiloxane cross-linker (7%).

**Fig. 1:**
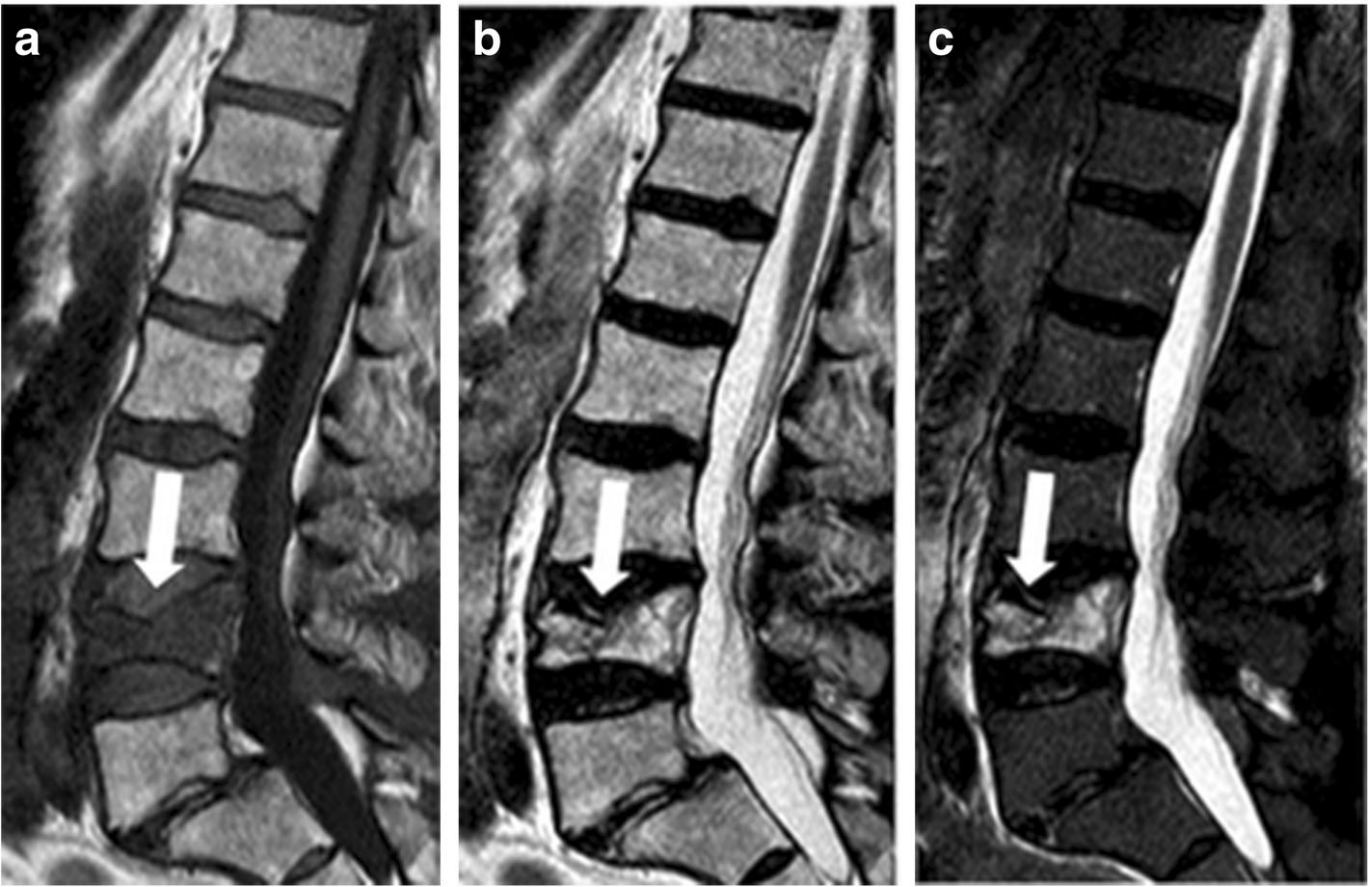
A 72-year old male/female patient with lumbar vertebral fracture treated with image-guided elastoplasty. MRI before treatment: sagittal T1-weighted (**a**), proton density-weighted (**b**), and T2-weighted (**c**) images. A lumbar vertebral fracture in L4 is clearly visualised as decreased signal of the fractured vertebral body in (**a**) (arrow, **a**) and increased signal in fractured vertebral body in (**b**) and (**c**) (arrow)

**Fig. 2:**
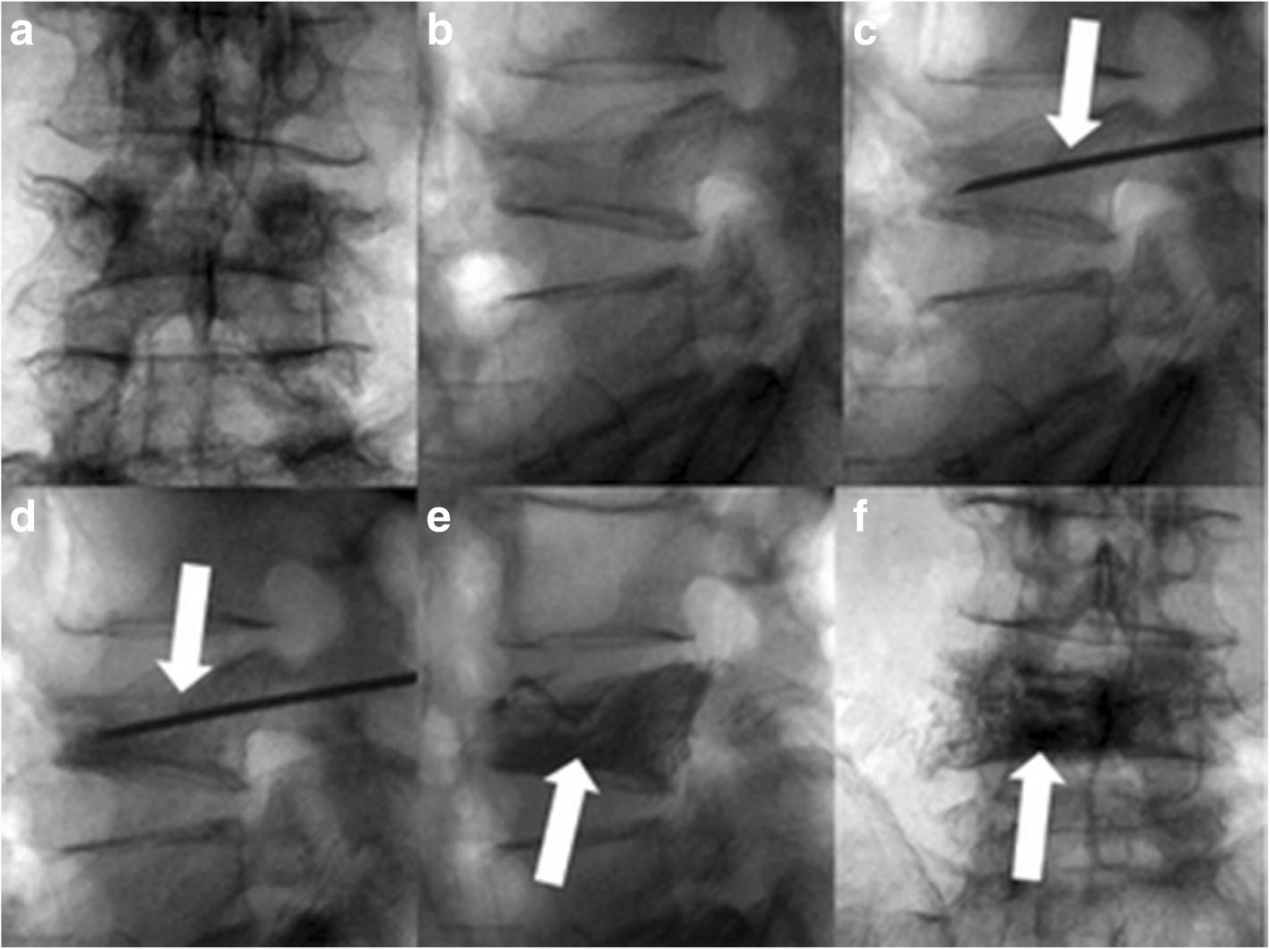
Same case as shown in Fig. 1. Elastoplasty procedure: injection of VK100 in the fractured body (L4) using the transpedicular approach under fluoroscopic guidance. Preprocedural control x-rays; anteroposterior (**a**) and sagittal (**b**) view; fluoroscopy during transpedicular needle insertion (arrow) showing correct placement of the needle into the vertebral body (**c**, **d**); postprocedural control x-rays showing the good filling of L4 body (arrow, **e**, **f**)

VK100 injection was stopped when satisfactory intervertebral material distribution was observed. When leakage outside the vertebral body was detected, the procedure was immediately interrupted and symptoms or modifications of the vital signs were checked. After some minutes, injection was cautiously restarted. If leakage was still present, the injecting needle was slightly moved or rotated and injection restarted. When VK100 injection was complete, the cannulas were withdrawn. Patients were discharged after 4–6 h of observation. A case is shown in Figs. 1 and 2.

### Endpoints and data analysis

Primary endpoints of the study were the technical success and safety of the procedure. Technical success was defined according to previously reported criteria for interventional procedures [12] as the ability of completing the treatment as preoperatively planned (i.e. achieving the desired filling of the vertebral body). Complications were recorded and classified according to the guidelines of the Society of Interventional Radiology [12]. Moreover, evidence of leakage during the procedure and presence of pulmonary emboli detected at post-procedural chest x-ray were recorded.

**Fig. 3:**
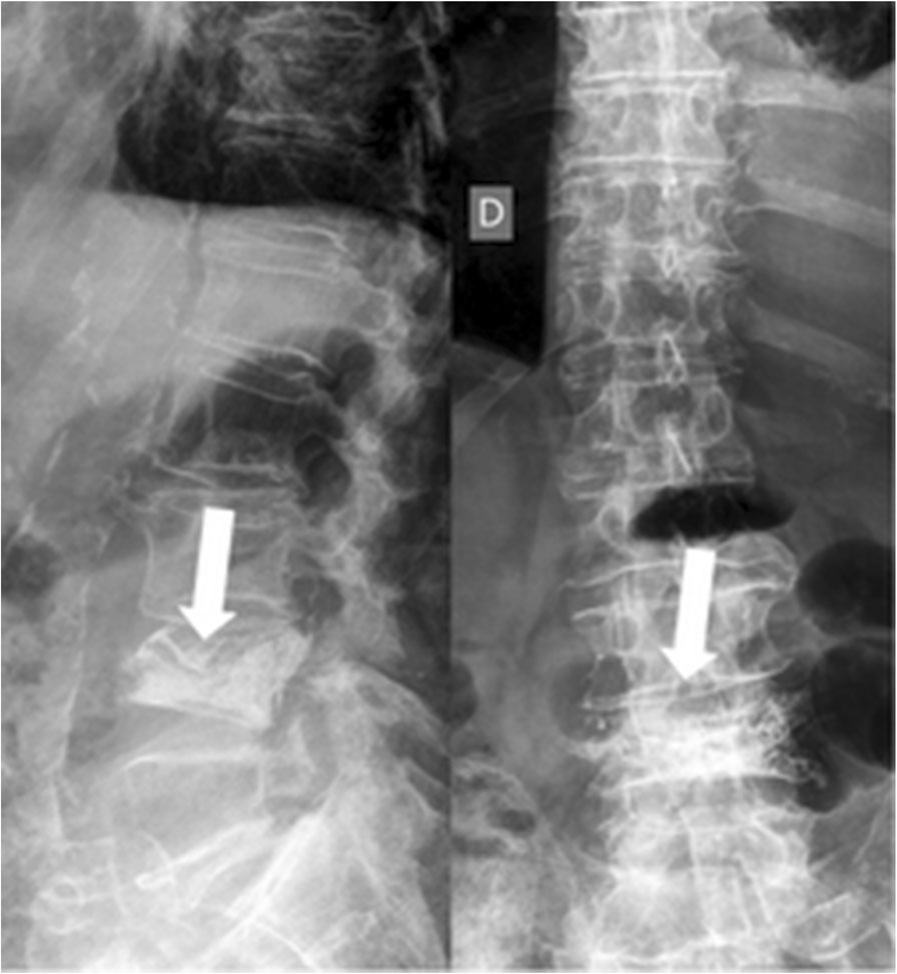
Same case as shown in Figs. 1 and 2. Lumbosacral spine x-ray examination performed one month after the elastoplasty procedure demonstrating the good filling of L4 vertebral body (arrows). No evidence of new vertebral fractures is seen

Secondary endpoints were the technical efficacy in achieving pain relief and the rate of secondary fractures during follow-up (Fig. 3). Visual analogue scale (VAS) was used to assess perceived pain before and after the procedure [13] while quality of life was assessed using the Oswestry Disability Index (ODI) [14] before and after the procedure. Procedure was regarded as effective if a reduction of > 2 points in the VAS scale and of > 15 percentage points in the ODI was obtained after the procedure.

All patients underwent a thoracic and lumbosacral spine x-ray examination and a clinical visit one month after the procedure. The number of patients who stopped to use a brace and number of patients who stopped using any medication for pain management were recorded. After the examination and discharge, patients were asked to immediately contact the interventional radiologist who performed the procedure if they experience any increase or restart of the back pain. In such cases, magnetic resonance imaging (MRI) or computed tomography (CT) of the spine were performed to asses for the presence of secondary fractures. Where no pain was reported by the patients, clinically relevant secondary fractures were excluded.

### Statistical analysis

VAS and ODI scores before and after the procedure were compared using the Mann-Whitney *U* test. The number of patients who needed a brace and pain medications before and after the treatment was compared using Fisher’s exact test. Analysis was performed using GraphPad Prism 5 software (Graph-Pad, La Jolla, CA, USA). A *p* value < 0.05 was considered significant.

## Results

In all cases it was possible to complete the procedure and to reach a satisfactory filling of the vertebral body (technical success 100%). No major complications occurred. In 6/19 patients (31.5%), a minimal leakage of the material was seen during the procedure. No change in the vital signs or symptoms were recorded in these cases. After a few minutes it was always possible to restart the procedure and conclude it as planned. None of these patients had modifications of the saturation parameters or symptoms after the procedure.

The procedure was effective in achieving an improvement in clinical symptoms in 18/19 patients (technical efficacy 94%). One patient with painful angioma did not experience any change in symptoms. VAS and ODI scores were significantly reduced after the procedure, from 7.9 ± 1.1 to 0.7 ± 1.4 (*p* < 0.001) and from 79.6 ± 12% to 9.9 ± 14% (*p* < 0.001), respectively. Of 15 patients, 14 (93%) no longer required a brace after the procedure (*p* < 0.001) and 16/19 (84%) completely stopped using any drugs for pain relief after treatment (*p* < 0.001).

At a mean follow-up time of 26.5 ± 28.1 months, no patients developed new symptoms or demonstrated a secondary fracture.

## Discussion

Vertebral augmentation has been reported to be highly effective in the relief of pain relief arising from vertebral lesions; it is nowadays becoming increasingly widespread in clinical practice. However, as already mentioned, one of the major controversies regarding the clinical application of vertebroplasty is the possible occurrence of secondary vertebral fractures due to an increased stiffness of the treated vertebral body [15]. In this scenario, the application of a more pliable material than PMMA [7] to achieve the vertebral stabilisation would be highly beneficial, theoretically lowering the number of secondary fractures.

The results of the present study play in favour of the effectiveness of elastoplasty with VK100 is in treating painful vertebral lesions, with a low rate of minor anticipated complications and no major complications. Moreover, no secondary fractures were found in our small series during follow-up. In our series, the vertebral augmentation procedure with VK100 was always feasible (technical success 100%). According to the subjective experience of the interventional radiologist, it was not more technically difficult than standard vertebroplasty with PMMA.

Practitioners have suggested that VK100 may cause an increased rate of leakage due to viscosity. However, viscosity is a moving target. During the curing of PMMA or the cross-linking of VK100, the viscosity is changing. Viscosity test data demonstrate that VK100 is equivalent to PMMA as used in vertebral augmentation. At an ambient temperature of 68–69 °F, it was shown that the average viscosity of VK100 at the time of injection into the vertebral body is 167,900 centipoise (cP) and the average viscosity of PMMA cements commonly used for vertebroplasty is 136,300 cP [16]. Further, VK100 is temperature-sensitive: as the temperature rises, the material cross-links at a much faster rate. At normal body temperature, it cross-links four times faster than at room temperature; once in the body, VK100 solidifies quickly and becomes too viscous to migrate. Moreover, unlike PMMA which breaks into small pieces that can travel in the body, VK100 strongly adheres to itself and bone, and when it is injected into the vertebrae it creates a single piece of silicone which sticks to the vertebral body.

The experience of the operator may play a relevant role in reducing the complication rate. Appropriate training should be used to achieve optimal results. In our series, all the procedures were performed by the same operator with more than 13 years of experience in vertebral augmentation procedures and no major complications occurred.

Minor leakage of VK100 outside the vertebral body was seen in about 30% of cases during the procedure. Material leakage has been reported to occur in vertebroplasty in a highly variable rate. In a systematic review of 15 studies, leakage of cement outside the vertebral body was reported in a range of 3.3–75.6% [17]. Rate of leakage and pulmonary embolism have been reported to be associated with the viscosity of the material used [18]. Thus, from our and previous experiences, elastoplasty seems to be similar to PMMA with respect to leakage.

One of the most critical complications related to extravertebral leakage of injected material is pulmonary embolism. This occurrence has been reported in a variable range for PMMA procedures [19–22]. Kim et al. [19] carried out a prospective study aimed at detecting incidence and understanding risk factor for pulmonary embolism in a group of 78 patients treated with cement vertebroplasty; they reported a rate of pulmonary embolism of about 23%. Urlings et al. [8] reported an extremely elevated rate of 60% of pulmonary embolism with the use of VK100, with one case of severe dyspnoea, and their results are totally different from our experience. One hypothesis to explain such a difference could be the different experience in performing vertebral augmentation in the two series, further underlying the crucial role of operator experience in vertebral interventional procedures. In our series, to avoid unnecessary radiation exposure, pulmonary CT could have been performed only in patients with respiratory symptoms such as dyspnoea. However, no patient presented respiratory symptoms after the procedure or at follow-up.

Regarding the efficacy in pain relief, in a systematic review on patients with osteoporotic vertebral compression fractures*,* mean pain scores, measured using a 0 to 10 VAS score, improved significantly from 7.8 to 3.1 (a 60.3% reduction) immediately after percutaneous vertebroplasty [23]. In our series, we achieved improvement in clinical symptoms in 94% of the cases, with a significant improvement in both VAS and ODI scores. Pain relief was noted almost immediately after the procedure, as happened in our clinical practice with standard vertebroplasty. Notably, 93% of patients previously carrying a brace were able to stop its use after elastoplasty and 84% of patients were able to completely stop the use of any pain relief drugs. Thus, our results in terms of pain relief are similar to those reported for standard vertebroplasty.

During the follow-up, no new vertebral fractures occurred in our series. In the literature, several studies reported on the development of new vertebral compression fractures in patients treated with vertebroplasty [24–26]. In a systematic review of the literature, Ma et al. [25] analysed 24 observational studies involving 3789 patients and found strong evidence for three risk factors associated with new vertebral fractures, including intradiscal cement leakage, lower bone mineral density and lower body mass index. On the other hand, the Vertos II study [27], a prospective multicentre randomised controlled trial comparing vertebroplasty and conservative therapy, showed no difference in the occurrence of new vertebral fractures between the two groups, but new vertebral fractures were observed in 16.4% of patients treated with vertebroplasty and in 24.7% of patients who underwent conservative therapy. Among the explanatory theories for new vertebral fractures correlated with vertebroplasty, some authors hypothesised an induced degenerative change in the adjacent bone due to an altered load transfer caused by the fact that cement-filled bone is much stiffer than cancellous bone [28–30]. In this scenario, the application of a material softer than PMMA has been suggested to reduce the stiffness of the treated vertebral body and consequently the risk of adjacent vertebral new fractures.

Some limitations of the present study need to be taken into consideration. First, this is a retrospective analysis on a limited number of patients treated over a long time period for different pathologies by a single operator with much experience in vertebral augmentation. These results might therefore not apply to the everyday clinical practice of any centre. Second, the presence of pulmonary embolism was evaluated only based on patient symptoms. This was due to the fact that we considered unjustified the radiation exposure associated with a chest CT in asymptomatic patients. Third, the occurrence of new vertebral fractures during follow-up has been only evaluated at one month with x-ray examinations and afterward only on the basis of clinical symptoms reported by the patients. Even if this might have determined an underestimation of new vertebral fractures, it should be underlined that the aim of the treatment is to solve the patient’s symptoms and that repeated radiological examinations in asymptomatic patients could be not justified.

In conclusion, elastoplasty with VK100 provided results in terms of pain relief similar to those reported for the most widely used vertebral augmentation techniques. The rate of minor complications was low and similar to that reported for other vertebral augmentation techniques. Finally, the absence of new vertebral fractures at one month after the procedure supports the rationale for applying elastic material for vertebral augmentation. However, even if the results from our preliminary experience are encouraging, further large prospective studies should be conducted to better define the role of elastoplasty among vertebral augmentation techniques.

## Abbreviations

ODI: Oswestry disability index
PMMA: Polymethylmethacrylate
VAS: Visual analogue scale
